# Time–Concentration Superposition for Linear Viscoelasticity of Polymer Solutions

**DOI:** 10.3390/polym15071807

**Published:** 2023-04-06

**Authors:** Can-Qi Li, Horst Henning Winter, Yuan-Qi Fan, Geng-Xin Xu, Xue-Feng Yuan

**Affiliations:** 1 Institute for Systems Rheology, Guangzhou University, No. 230 West Outer Ring Road, Higher Education Mega-Center, Panyu District, Guangzhou 510006, China; 2 Chemical Engineering and Polymer Science & Engineering, University of Massachusetts Amherst, Silvio O. Conte National Center for Polymer Research, 120 Governors Drive, Amherst, MA 01003-3110, USA

**Keywords:** time–concentration superposition principle, viscoelastic properties, polymer solutions

## Abstract

The concentration dependence of linear viscoelastic properties of polymer solutions is a well-studied topic in polymer physics. Dynamic scaling theories allow qualitative predictions of polymer solution rheology, but quantitative predictions are still limited to model polymers. Meanwhile, the scaling properties of non-model polymer solutions must be determined experimentally. In present paper, the time–concentration superposition (TCS) of experimental data is shown to be a robust procedure for studying the concentration scaling properties of binary and ternary polymer solutions. TCS can not only identify whether power law scaling may exist or not, and over which concentration range, but also unambiguously estimate the concentration scaling exponents of linear viscoelastic properties for a range of non-model polymer solutions.

## 1. Introduction

A polymer solution consists of a molecularly dispersed polymer in a low molecular weight solvent. Above a critical concentration c*, the macromolecules begin to interact just like in a polymer melt, but at much higher mobility. In the ideal case of monodisperse and sufficiently long flexible chain polymers, polymer solutions exhibit viscoelastic properties which are universal [1,2,3]. For such model polymer solutions, under isobaric conditions, two state variables such as polymer concentration and solvent quality are sufficient to define their properties at equilibrium or quasi-equilibrium. Solvent quality is a measure of polymeric chain segment–segment interactions mediated through a solvent. In the dilute limit, the mean radius of gyration of a molecular chain is scaled with the molecular weight, *M*, as $R_{g}\sim M^{\nu }$, where *ν* is a characteristic scaling exponent; 0.50 in θ solvents, and 0.588 in good solvents. The longest relaxation time is scaled as $\lambda \approx \lambda_{0}M^{\delta }$, where $\lambda_{0}$ is the relaxation time of a Kuhn monomer, δ=3ν is the Zimm model accounting for hydrodynamic interactions and δ=2 is the Rouse model for eligible hydrodynamic effects. The terminal modulus and the zero shear viscosity are scaled with the molecular weight and polymer concentration *c*, as $G_{c}\sim M^{-1}c$ and $\eta_{0}=G_{c}\lambda \sim M^{\delta -1}c$. All flexible chain polymers would follow such a universal scaling law, no matter how different in their chemical monomers. The complicity of polymer segment–segment thermodynamic and hydrodynamic interactions mediated with solvents is casted into a simple scaling theory, which captures the fundamental physics of polymer solutions very well.

The conformations of individual polymer chains start to overlap at an increased concentration, starting at $c^{*}\sim \frac{M}{R_{g}^{3\nu }}=M^{1-3\nu }$, above which the solutions are in the semidilute concentration range. Polymer dynamics in a semidilute solution involve multiple length and time scales and become much more complicated than those in a dilute solution. The polymer structure is characterized by its correlation length $\xi \sim c^{-\nu /\left(3\nu -1\right)}$ [2]. Below the correlation scale, the structure and dynamic properties exhibit the characteristics of dilute solutions. On a larger scale, the interchain interactions are screened, and the dynamic properties of polymer solutions behave like those of polymer melts. The properties of an unentangled polymer in semidilute solutions could be predicted by the Rouse model as $\lambda \sim M^{2}c^{\left(2-3\nu )/(3\nu -1\right)}$, $G_{c}\sim M^{-1}c$ and $\eta_{0}\approx G_{c}\lambda \sim M^{-1}c^{1/(3\nu -1)}$. For monodisperse entangled polymer solutions, the reptation model predicts $\lambda \sim M^{3}c^{3(1-\nu )/(3\nu -1)}$, $G_{c}\sim c^{3\nu /(3\nu -1)}$ and $\eta_{0}\approx G_{c}\lambda \sim c^{3/(3\nu -1)}$ [3]. These classical relations were derived for model polymers with linear flexible chains of uniform and infinite length. The scaling exponent depends on concentration range and solvent quality as could be validated by experiments with a range of model polymer solutions [4]. Again, the dynamic theory gives reasonably good predictions for the rheological properties of concentrated polymer solutions in which polymer chains are well entangled. However, those dynamic scaling theories cannot account for many practical factors deviated from the model polymer fluids such as finite length of a polymer chain, polydispersity, variation of solvent quality between a good solvent and θ-solvent, ionic strength, possible phase transitions in different temperatures and concentrations, etc. (hereafter called non-model polymers). This poses the question: Do solutions of non-model polymers also exhibit a well-defined power law concentration dependence, especially over semidilute and entangled concentration regions? If so, how can the concentration scaling exponents be estimated in an unambiguous way despite of all the complicated factors involved?

This paper focuses on the concentration scaling analysis of linear viscoelasticity of non-model polymer solutions above their overlap concentration, c/c* > 1, semidilute entangled and concentrated solutions. Frequency-dependent, dynamic moduli *G*′ and *G*″ from small amplitude oscillation shear (SAOS) experiments are analyzed using time–concentration superposition (TCS). As known from time–temperature superposition (TTS) [5], the shifting of *G*′ and *G*″ data can be separated in a frequency shift *a*_T_ and a modulus shift *b*_T_, which has its equivalent, now, in *a*_c_ and *b*_c_ for TCS. We will determine the concentration shift factors a_c_ (c/c*) and *b_c_* (*c*/c*) in search of an unambiguous way to estimate the concentration scaling exponents of non-model polymer solutions in comparison to the universal properties known for model polymer solutions. The shift factor of the complex viscosity, *b_c_*/*a_c_*, is also included. Equivalently to TTS, TCS is shown to expand the experimental frequency window of the SAOS data. This has been studied experimentally with a typical non-model polymer solution. The results were compared to data available from other laboratories that have not yet been analyzed in this way.

## 2. Materials and Methods

### 2.1. Polymer Solution

A sample of commercially available polyacrylamide (PAAm) with a nominal average molecular weight (*M_w_*) of 18 × 10^6^ g/mol was purchased from Polysciences, Inc. (Catalogue No.: 18522-100), Warrington, PA, USA, and used as received (hereafter called 18M PAAm). A series of ternary solutions containing 18M PAAm, deionized water and 60 *wt.*% sucrose (hereafter called 18M PAAm–sucrose–water solution) with a concentration range from 0.173 *wt*.% to 0.921 *wt*.% were prepared by firstly dissolving 18M PAAm powder in deionized water, obtained from the Direct-Q UV-R pure water system after three stages of treatment at room temperature, and then adding an adequate amount of sucrose, purchased from Chemical Technology Co., Ltd. (Catalogue No.:M20224), Meryer (Shanghai), China to make up 60 *wt.*% sucrose and gently stirring at room temperature using an IKA C-MAG HS 7 magnetic stirrer at 12 rpm for 72 h. The ternary solutions were then put into a 4 °C refrigerator for further homogenization and storage.

Light scattering measurement of dilute PAAm in water solution reported by Francois et.al. [6] gives the average radius of gyration scaled with molecular weight (*M*) as $R_{g}=0.0749M_{w}^{0.64\pm 0.01}$(Å) with a power law exponent exceeding 0.588; therefore, water is considered a super-good solvent for PAAm. The molecular weight dependence of zero-gradient intrinsic viscosities for the same sample is approximately scaled with molecular weight as $[\eta ]=(9.3\times 10^{-3})M_{w}^{0.75\pm 0.01}$. Accordingly, the radius of gyration of the sample used here could be estimated as $R_{g}=3295$(Å). Using a simple cubic packing assumption, the overlap concentration was estimated as c* = 0.00946 *wt.*% (approximately considered as 0.01 *wt.*% hereafter). Hence, the 18M PAAm–sucrose–water solutions with the concentration range from 17.3 c* to 92.1 c* were characterized here. Further note that due to a lack of the standard high-molecular-weight polymer sample, quantitative characterization of the molecular weight and distribution of the 18M PAAm sample using Gel Permeation Chromatography is very difficult if not impossible. However, it may be reasonable to expect that its polydispersity index is significantly higher than that of the 5M PAAm sample, having *M_w_* = 5.7 × 10^6^ g/mol and *M_w_*/*M_n_* = 34.4, supplied by the same source and characterized in our previous work [7].

### 2.2. Rheometry

An ARES-G2 shear rheometer (TA Instruments) with a cone-plate fixture (D = 50 mm, a = 0.04 rad) was used for rheological characterization. The experimental temperature 25 °C was controlled with an Advanced Peltier System which connected to a ThermoCube Model 10-300 (115/230 V, 50/60 Hz) water bath system with a temperature control accuracy of ±0.2 K.

The 18M PAAm–sucrose–water solutions were first subjected to a set of oscillatory amplitude experiments prior to SAOS experiments in order to determine the strain range in the linear viscoelastic region and maintain a consistent strain history. SAOS experiments were then performed to characterize the linear rheological properties of the 18M PAAm–sucrose–water solutions. The results are analyzed in comparison with the concentration scaling of other binary polymer solutions.

## 3. Results

**Figure 1:**
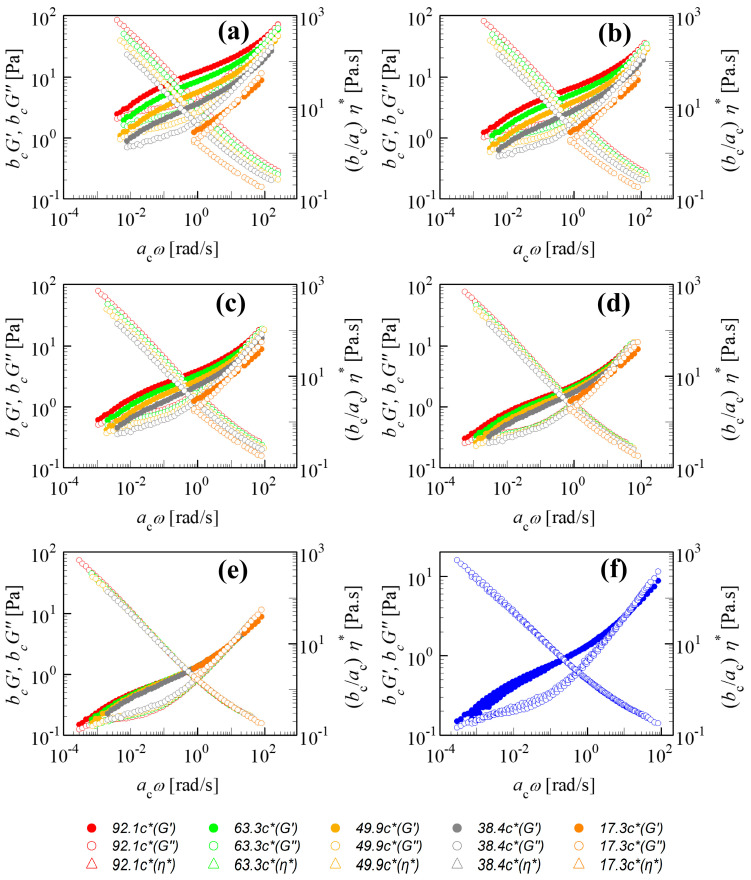
Time–concentration superposition (TCS) of SAOS data belonging to a 18M PAAm–sucrose–water solution at semidilute concentrations from 17.3c* to 92.1c*. TCS merges the linear viscoelastic data *G*′, *G*″, η*(ω, c/c*) thereby generating master curves at the reference concentration of 17.3c* as measured by SAOS. (**a**) Original data before shifting; (**b**) 25% shifting towards the reference state; (**c**) 50% shifting the curves; (**d**) 75% shifting the curves; (**e**) 100% shifting the curves; (**f**) a master curve of *G*′(ω, c/c*), *G*″(ω, c/c*), η*(ω, c/c*) after completion of the TCS procedure. The shifting and plotting were executed using IRIS Rheo-hub software [8].

Linear viscoelastic properties of polymer solutions are strongly affected by the level of polymer loading as exemplified by the 18M PAAm–sucrose–water solution shown in Figure 1a. At high polymer concentrations, the internal dynamics slow down and the dynamic moduli *G*′(*ω*, c/c*), *G*″(*ω*, c/c*) increase their value. The slowing of characteristic times is not visible immediately because of the fixed frequency range. It requires the time–concentration shifting with its increased width of the frequency window due to gains from the additional slow modes in the highly concentrated solution samples. Such time–concentration superposition, if possible, merges all data into a single pair of dynamic moduli *G*′(*ω*, c_ref_/c*), *G*″(*ω*, c_ref_/c*). They belong to the solution at reference composition c_ref_/c*, abbreviated as *G′_ref_*, *G″_ref_* in the following.

Figure 1a–f demonstrate the TCS shifting for the linear viscoelastic properties of the 18M PAAm–sucrose–water solution with respect to Rouse dynamics. The unshifted experimental data define the starting condition for the shifting, as in Figure 1a. The lowest concentration *c_ref_* = 17.3c* was chosen here to serve as a reference state providing the frequency-dependent *G′_ref_* and *G″_ref_* in the relatively narrow frequency window of the SAOS experiment. These values for *c_ref_* = 17.3c* are fixed during the shifting while the higher *c*/c* moduli become shifted to the left and down thereby widening the frequency window. Stepwise progress of the shifting is pictured as a sequence. When the shifting is complete, the rescaled moduli superimpose on *G′_ref_* and *G″_ref_* of the reference state and a set of master curves is generated. These master curves belong to *c_ref_* = 17.3c* but can now be shifted to other concentrations.

The shift direction depends on a solution’s concentration with respect to the reference state: for *c*/*c_ref_* < 1, *b*_c_ is positive and *a*_c_ is negative and for *c*/*c_ref_* > 1, the signs change and *b*_c_ < 1 and *a*_c_ > 1. Since TCS in Figure 1 is based on the lowest concentration, all shifting occurs to one side.

**Figure 2:**
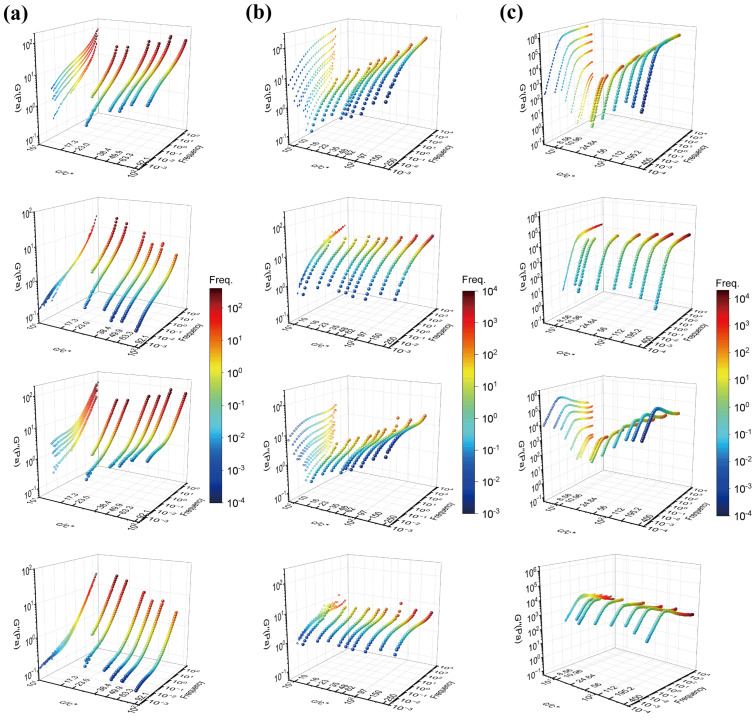
Three–dimensional plots of *G*′ and *G*″ against concentration and frequency. (**a**) 18M PAAm–sucrose–water ternary solution; (**b**) 18M PAAm–water solution [11]; and (**c**) PB–PHO solution [4], respectively. The first row and the third row show the curves before TCS shifting. The second row and the fourth row show the curves after TCS shifting. The color scheme indicates the scale of frequency. The master curves are projected in the *G*′– or *G*″–Freq plane.

The shifted *G*′ and *G*″ are not so well superimposed in the low-frequency region. This has commonly been observed in the other polymer solutions [9,10,11,12,13]. Figure 2 shows three-dimensional plots of *G*′ and *G*″ against concentration and frequency for the 18M PAAm–sucrose–water ternary solution, the 18M PAAm–water binary solution [11] and the monodisperse polybutadiene (PB) in phenyloctane (PHO) binary solution [4] before and after TCS shifting. The three-dimensional curves are also projected on the G′- or G″-Freq plane of the three-dimensional plots in order to show the outcomes of TCS superposition clearly. As shown in Figure 2a, it is evident that there is a frequency window, either in the frequency region or the low-frequency region, over which the concentration scaling of *G*′ and *G*″ exhibits a dynamic similarity with the same power law exponent. However, the scaling exponents are significantly different between the high-frequency region and the low-frequency region, indicating the existence of different dynamic mechanisms corresponding to Rouse dynamics and terminal entanglement dynamics, respectively. Similarly, as shown in Figure 2b,c, the terminal entanglement dynamics dominate over a much wider frequency window, which extends several orders of the magnitude for the 18M PAAm–water solution and the PB–PHO solution.

**Figure 3:**
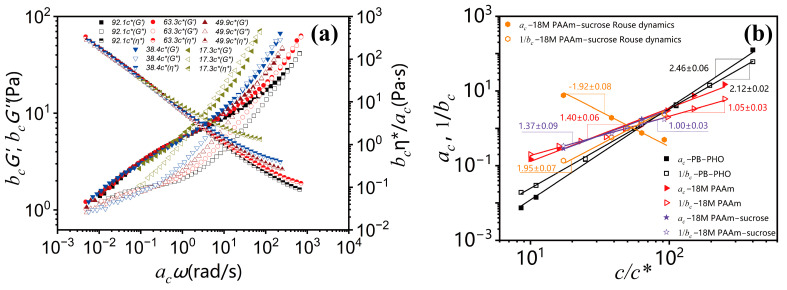
(**a**) A master curve of *G*′(ω, c/c*), *G″*(ω, c/c*) complex viscosity η*(ω, c/c*) with *c_ref_* = 49.9c* superimposed with respect to the terminal entanglement dynamics; (**b**) plots of shifting factor *a*_c_ and 1/*b*_c_ against concentration *c*/c* and in comparison with PB–PHO [4] and 18M PAAm–water binary solutions, respectively.

However, by closely examining those *G*′ and *G*″ curves in the relatively high frequency, it is also evident that the data are not perfectly superimposed. The apparent superposition discrepancy simply reflects the fact that dynamics with well-separated time and length scales would follow different dynamic similarity, respectively, in terms of concentration scaling. As long as the *G′* and *G″* data with various concentrations could be superimposed over a certain range of frequency (no matter how narrow it would be), the dynamic similarity would hold under the corresponding dynamics with its own characteristic time and length scale. The superposition discrepancy in other dynamics over different time and length scales is irrelevant to the dynamics being considered. According to the dynamic scaling theory [1,2,3], the characteristic times of Rouse dynamics are well separated from the characteristic time of the entanglement dynamics, and the ratio between the reptation time and the Rouse time is proportional to the length of the polymer chain. Therefore, the concentration scaling of Rouse dynamics should also be different from the terminal flow dynamics dominated by the entanglement dynamics. As such, TCS could only be realized either in the Rouse dynamic regime or in the terminal entanglement dynamic regime. Alternatively, the same linear viscoelastic data of Figure 1a and Figure 2a could also be superimposed with respect to the terminal dynamic regime. The results are shown in Figure 3a. Note that due to the same reason, the better superimposed linear viscoelastic data of the 18M PAAm–sucrose–water ternary solutions over the terminal dynamic regime would inevitably result in the less well superimposed high-frequency data. Although the outcome of TCS might not look perfect, the resulting shift factors could be related to a power law concentration scaling as

(1) $${a_{c}(c;c_{ref})=a_{c}{(c)/a}_{c}{(c}_{ref})=\left(\frac{c}{c_{ref}}\right)}^{\alpha },$$

(2) $${b_{c}(c;c_{ref})=b_{c}{(c)/b}_{c}{(c}_{ref})=\left(\frac{c}{c_{ref}}\right)}^{\beta }.$$

The concentration scaling of the shifting factors *a*_c_ is equivalent to the concentration scaling of either the terminal relaxation time or the Rouse relaxation time, respectively. The time–concentration shift effectively amounts to a multiplication of the dynamic moduli *G*′(*ω*, *c*) and *G*″(*ω*, *c*) with the above modulus shift factor *b_c_* and a multiplication of the experimental frequency with the above *a_c_* as

(3) $$b_{c}G^{\prime }\left(a_{c}\omega ,c/c^{*}\right)={G^{\prime }}_{ref},$$

(4) $$b_{c}G^{\prime \prime }\left(a_{c}\omega ,c/c^{*}\right)={G^{\prime \prime }}_{ref}.$$

The concentration shifting also applies to the complex viscosity. It superimposes the viscosity data (any concentration within range) onto a reference viscosity function:

(5) $$\eta_{ref}^{*}=\eta^{*}\left(a_{c}\omega ,c_{ref}/c^{*}\right).$$

Typical viscosity shifting is shown in Figure 1b–f and Figure 3a. Shifting involves rescaling of complex viscosity data:

(6) $$\frac{b_{c}}{a_{c}}\eta^{*}\left(a_{c}\omega ,c/c^{*}\right)=\eta_{ref}^{*}.$$

The viscosity shifting gains from both shift factors, *a*_c_ and *b*_c_, and hence is larger than the shifting of *G*′ or *G*″ as

(7) $$\frac{\eta^{*}\left(a_{c}\omega ,c/c^{*}\right)}{\eta_{ref}^{*}}=\frac{a_{c}}{b_{c}}={\left(\frac{c}{c_{ref}}\right)}^{\alpha -\beta }.$$

Obviously, as long as a power law concentration scaling on linear viscoelastic properties of polymer solutions exists, TCS is a robust way to estimate their scaling exponents.

**Figure 4:**
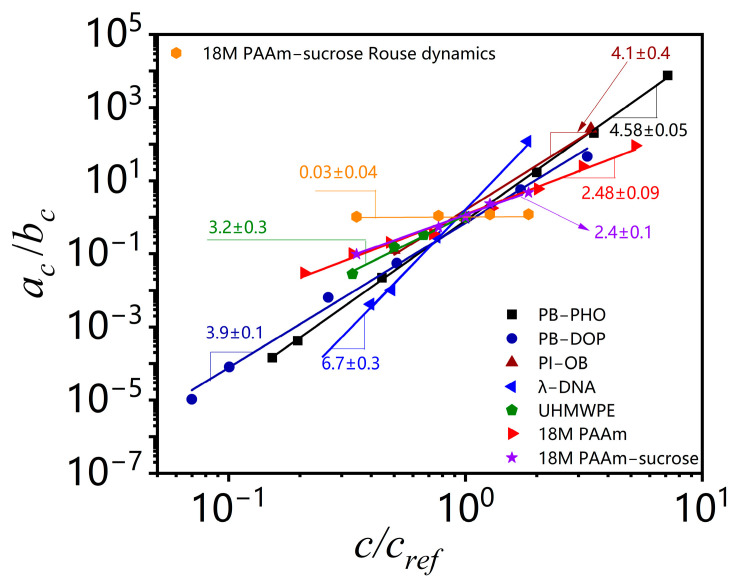
Plots of (*a*_c_/*b*_c_) as a function of (*c*/*c_ref_*) in the logarithmic scales for the polymer solutions listed in Table 1 with respect to reference concentration as *c_ref_* = 56c* for PB–PHO [4], *c_ref_* = 44.3c* for PB–DOP [4], *c_ref_* = 8.0c* for PI–OB [13], *c_ref_* = 49.1c* for λ-DNA [9], *c_ref_* = 125c* for UHMWPE [12], *c_ref_* = 48c* for 18M PAAm aqueous solution [11], and *c_ref_* = 49.9c* for 18M PAAm–sucrose–water solution, respectively.

As shown in Figure 3b, the concentration scaling of the horizontal shifting factor *a*_c_ and the vertical shifting factor *b*_c_ over the terminal entanglement dynamics and Rouse dynamics for 18M PAAm–sucrose–water ternary solutions is compared with the concentration scaling of the terminal entanglement dynamics for 18M PAAm–water binary solution [11] and monodisperse PB–PHO binary solution [4]. The concentration scaling of the 18M PAAm–water ternary solutions over the terminal entanglement dynamics is in excellent agreement with those of the 18M PAAm–water binary solutions. It shows that the solvent quality of 60 *wt.*% sucrose and water mixture is very similar to that of pure water. However, the power law concentration scaling exponent of the shifting factor *a*_c_ over Rouse dynamics shows a negative sign. It reflects the fact that the characteristic relaxation time over Rouse dynamics is decreased with the increase in polymer concentration, likely due to the screening effect of segment–segment interactions in polymer solutions. The estimated concentration scaling exponents α and β for PB–PHO, 18M PAAm binary and ternary solutions are listed in Table 1 along with the results of other binary polymer solutions obtained from the TCS procedure over the terminal entanglement dynamic regime. The concentration scaling of complex viscosity is equivalent to the scaling of the shifting factors *a*_c_/*b*_c_. As shown in Figure 4, they all exhibit a well-defined power law concentration scaling. Table 1 also lists the estimated concentration scaling exponent (*α* − *β*) for all the polymer solutions analyzed here. The convolution of shift factors in the viscosity shift obscures its meaning and makes it hard to draw conclusions about concentration effects on other viscoelastic material functions.

## 4. Discussion

There is still considerable ambiguity in predicting the power law concentration scaling exponent of linear viscoelastic properties of entangled polymer solutions. Under single-parameter scaling approximation proposed by de Gennes [2] and the scaling of the zero shear viscosity with molecular weight as $\eta_{0}\sim M^{3.4}$, the single-parameter scaling theory predicts $\eta_{0}\sim c^{6.8}$ for polymer in θ solvent solution and $\eta_{0}\sim c^{4.5}$ for polymer in good solvent solution. On the other hand, under the two-parameter scaling approximation [14], which accounts for a possible difference in concentration dependence of the tube diameter and the correlation length, the two-parameter scaling theory estimates the concentration scaling exponents of the zero shear viscosity for θ solvent as 5.2 and good solvent polymer solutions as 4.5, respectively. The same scaling exponent (4.5) of the zero shear viscosity is found for good solvent PB–PHO solutions and θ solvent PB–DOP solutions [4]. Although the dynamic scaling theory could give a reasonably consistent prediction to the concentration scaling of the zero shear viscosity of the above model polymer solutions and the results are also in quite good agreement with those estimated by the TCS procedure, there are still significant discrepancies in the estimated concentration scaling exponent of the reptation time and terminal modulus.

Table 1 lists the outcomes of the TCS analysis for a range of non-model polymer solutions, along with the model polymer solutions. The results show that the concentration scaling is very sensitive to the actual dependence of correlation length, tube diameter, reptation time, terminal modulus, and viscosity on solvent quality, finite molecular length and polydispersity, possible changes of equilibrium phase or thermodynamic state with concentration at a certain temperature, etc. Under a marginal solvent quality between good and θ conditions, the concentration scaling of the zero shear viscosity for PI–OB solutions sets between good solvent PB–PHO solution and θ solvent PB–DOP solutions. The scaling exponents of its terminal entanglement time and terminal modulus are out of the range between the two limits defined by PB–PHO and PB–DOP solutions. The concentration scaling of Welan solutions is similar to that of PI–OB solutions. The effect of polymer chain length is evident, in addition to solvent quality. The effect is much more pronounced to the concentration scaling of the terminal entanglement time for completely monodisperse ultrahigh molecular weight λ-DNA, resulting in a higher scaling exponent of the zero shear viscosity. The different Kuhn length and finite size of those polymers might also be important factors that cause different concentration scaling. TCS results of highly polydisperse UHMWPE, 18M PAAm binary and ternary solutions also show a general trend that the polydispersity could significantly reduce the concentration scaling exponents of the terminal entanglement time and the zero shear viscosity. At present, no unified theory can quantitatively account for all the effects deviated from the model polymer solutions. The present work illustrates that TCS could implicitly account for all the above effects for non-model as well as model polymer solutions and also for colloidal suspensions, as reported in a recent study that the power law concentration scaling exponent depends on colloidal interactions mediated with solvent and is sensitive to equilibrium phase behavior of colloidal suspensions [15].

## 5. Conclusions

The concentration shift was analyzed using the same framework as JD Ferry’s time–temperature superposition. Surprisingly, power law scaling was found far above the overlap concentration. Unlike time–temperature superposition, both shifts are substantial, with the vertical shift sometimes being as large as the time shift, or even larger. The data analysis presented here indicates that TCS is a powerful alternative method to estimate the concentration scaling of linear viscoelastic properties of polymer solutions. It can account for actual solvent quality, flexibility of the molecular chain, molecular weight, and distribution. It can also identify the concentration range where power law scaling may or may not exist and estimate the concentration scaling exponents of linear viscoelastic properties unambiguously. Moreover, the dependence of scaling exponents over the concentration region indicates possible different dynamic similarity and corresponding structural features, e.g., from the Zimm dynamics for dilute polymer solutions, the Rouse dynamics for concentrated but unentangled polymer solutions, to the reptation dynamics for highly entangled polymer solutions and possible phase transitions of liquid crystalline polymer solutions. Hence, this method is significant in predicting and optimizing engineering for non-model polymer solutions.

The relatively “clean” exponent values of *α* ≈ 5/2 and *β* ≈ −2 for monodisperse PB–PHO solutions, *α* ≈ 3/2 and *β* ≈ −1 for highly polydisperse 18M PAAm aqueous solutions, and *α* ≈ −2 and *β* ≈ −2 for highly polydisperse 18M PAAm–sucrose–water solutions must be considered coincidental at this level of information. Further experiments are needed to explore the generality of these values for classes of polymer solutions with shared characteristics such as solvent quality, molecular interaction, distance from any type of equilibrium phase transition, etc. This framework provides a starting point for such exploration.

## Figures and Tables

**Table 1:** The concentration scaling exponents *α*, −*β* and (*α* − *β*) of the shifting factors *a*_c_, 1/*b_c_* and *a*_c_/*b*_c_, respectively, are estimated by TCS for a range of polymer solutions. PB–PHO and PB–DOP are monodisperse polybutadiene (PB) in phenyloctane (PHO) and in dioctyl phthalate (DOP) [4], respectively; PI–OB is monodisperse *cis-*polyisoprene (PI) in oligobutadiene (OB), which is a marginal solvent between good and theta solvent [13]; λ-DNA is completely monodisperse ultrahigh molecular weight linear lambda (λ) DNA in Tris-EDTA buffer [9]; UHMWPE is ultrahigh molecular weight polyethylene dissolved in oligo-ethylene [12]; Welan gum solutions but no information on molecular characterization available [10]; 18M PAAm is highly polydisperse 18M PAAm in aqueous binary solution [11] and in 60 *wt.%* sucrose–water ternary solution, respectively. The 18M PAAm ternary solution was analyzed with respect to the terminal entanglement dynamics as well as Rouse dynamics in which the exponent *α* shows a negative value.

Sample Code	*M_w_* (g/mol)	*M_w_*/*M_n_*	c/c*	Solvent Quality	*α*	−*β*	*α* − *β*
PB–PHO [4]	9.25 × 10^5^	<1.1	8.56~400	Good	2.46 ± 0.06	2.12 ± 0.02	4.58 ± 0.05
PB–DOP [4]	9.25 × 10^5^	<1.1	3.1~144.9	θ	1.8 ± 0.1	2.14 ± 0.03	3.9 ± 0.1
PI–OB [13]	4.88 × 10^4^	1.05	4~27.1	Marginal between good and θ solvent	2.9 ± 0.4	1.19 ± 0.05	4.1 ± 0.4
λ-DNA [9]	3.15 × 10^7^	1.0	19.5~89.7	Good	4.7 ± 0.3	2.0 ± 0.1	6.7 ± 0.3
UHMWPE [12]	3.2 × 10^6^	9.7	41.7~125	θ	1.0 ± 0.2	2.2 ± 0.1	3.2 ± 0.3
Welan [10]	-	-	-	-	3.6 ± 0.2	1.16 ± 0.01	4.8 ± 0.2
18M PAAm [11]	18 × 10^6^	>34.4	10~250 in water	Super-good	1.40 ± 0.06	1.05 ± 0.03	2.48 ± 0.09
			17.3~92.1 in 60 *wt.*% sucrose–water		1.37 ± 0.09	1.00 ± 0.04	2.4 ± 0.1
					−1.92 ± 0.08	1.95 ± 0.07	0.03 ± 0.04

## Data Availability

The data presented in this study are available on request from the corresponding authors.

## References

[1] Colby R.H. (2010). Structure and linear viscoelasticity of flexible polymer solutions: Comparison of polyelectrolyte and neutral polymer solutions. Rheol. Acta.

[2] De Gennes P.G. (1979). Scaling Concepts in Polymer Physics.

[3] Doi M., Edwards S.F. (1986). The Theory of Polymer Dynamics.

[4] Colby R.H., Fetters L.J., Funk W.G., Graessley W.W. (1991). Effect of concentration and thermodynamic interaction on the viscoelastic properties of polymer solutions. Macromolecules.

[5] Ferry J.D. (1980). Viscoelastic Properties of Polymer.

[6] Francois J., Schwartz T., Weill G. (1980). Crossover from theta to the excluded volume single chain statistics: New experimental evidences and a modified blob model. Macromolecules.

[7] Lanzaro A., Yuan X.-F. (2011). Effects of contraction ratio on non-linear dynamics of semi-dilute, highly polydisperse PAAm solutions in microfluidics. J. Non-Newton. Fluid Mech..

[8] Poh L., Narimissa E., Wagner M.H., Winter H.H. (2022). Interactive Shear and Extensional Rheology—25 years of IRIS Software. Rheol. Acta.

[9] Banik S., Kong D., Francisco M.J.S., McKenna G.B. (2021). Monodisperse lambda DNA as a model to conventional polymers: A concentration-dependent scaling of the rheological properties. Macromolecules.

[10] Carmona J.A., Ramírez P., García M.C., Santos J., Muñoz J. (2019). Linear and non-linear flow behavior of welan gum solutions. Rheol. Acta.

[11] Fan Y.-Q., Lanzaro A., Yuan X.-F. (2022). Universal concentration scaling on rheometric properties of polydisperse and high molecular weight polyacrylamide aqueous solutions. Chin. J. Polym. Sci..

[12] Ianniello V., Costanzo S., Pasquio R., Ianniruberto G., Troisi E., Tervoort T.A., Grizzuti N. (2022). Determination of the molecular weight distribution of ultrahigh molecular weight polyethylene from solution rheology. J. Rheol..

[13] Watanabe H., Yao M.-L., Osaki K. (1996). Comparison of dielectric and viscoelastic relaxation behavior of polyisoprene solutions: Coherence in subchain motion. Macromolecules.

[14] Colby R.H., Rubinstein M. (1990). Two-parameter scaling for polymers in Θ solvents. Macromolecules.

[15] Xu G.-X., Yuan X.-F., Liu Q.-S., Wang H. (2023). Concentration Scaling on Linear Viscoelastic Properties of Cellular Suspensions and Effects of Equilibrium Phase Behavior. Int. J. Mol. Sci..

